# Characterizing lesion morphology of a novel diamond-tip temperature-controlled irrigated radiofrequency ablation catheter

**DOI:** 10.1007/s10840-023-01595-9

**Published:** 2023-06-21

**Authors:** Tarvinder S. Dhanjal, Megan M. Schmidt, Michael K. Getman, Renee C. Brigham, Jaffar Al-Sheikhli, Ian Patchett, Melissa R. Robinson

**Affiliations:** 1 University Hospitals Coventry and Warwickshire, Clifford Bridge Road, Walsgrave, Coventry, CV2 2DX UK; 2 University of Warwick, Coventry, UK; 3 Medtronic, Inc., Minneapolis, MN USA; 4 University of Minnesota, Minneapolis, MN USA; 5 Samaritan Health Services, Corvallis, OR USA

**Keywords:** Atrial fibrillation, Catheter ablation, DiamondTemp, Ventricular lesions

## Abstract

**Background:**

The DiamondTemp ablation (DTA) system is a novel temperature-controlled irrigated radiofrequency (RF) ablation system that accurately measures tip-tissue temperatures for real-time power modulation. Lesion morphologies from longer RF durations with the DTA system have not been previously described. We sought to evaluate lesion characteristics of the DTA system when varying the application durations.

**Methods:**

A bench model using porcine myocardium was used to deliver discrete lesions in a simulated clinical environment. The DTA system was power-limited at 50 W with temperature set-points of 50 °C and 60 °C (denoted Group_50 and Group_60). Application durations were randomized with a range of 5–120 s.

**Results:**

In total, 280 applications were performed. Steam pops were observed in five applications: two applications at 90 s and three applications at 120 s. Lesion size (depth and maximum width) increased significantly with longer applications, until 60 s for both Group_50 and Group_60 (depth: 4.5 ± 1.2 mm and 5.6 ± 1.3 mm; maximum width: 9.3 ± 2.7mm and 11.2 ± 1.7mm, respectively). As lesions transition from resistive to conductive heating (longer than 10 s), the maximum width progressed in a sub-surface propagation. Using a “Time after Temperature 60 °C” (TaT_60_) analysis, depths of 2–3 mm occur in 0–5 s and depths plateau at 4.6 ± 0.8 mm between 20 and 30 s.

**Conclusions:**

The DTA system rapidly creates wide lesions with lesion depth increasing over time with application durations up to 60 s. Using a TaT_60_ approach is a promising ablation guidance that would benefit from further investigation.

## Introduction

Over the past three decades, radiofrequency (RF) catheter ablation has become a prominent method of treating cardiac arrhythmias [1, 2]. Early research focused on the RF biophysics of lesion formation and firmly established the therapeutic temperature range between irreversible tissue damage (> 50 °C) and the prevention of interstitial steam formation (< 100 °C) [3–9]. Since this time, catheter designs have evolved. Particularly, open irrigation and the inclusion of force sensors have facilitated more efficient lesion formation and improved safety. While improvements have been made, lesion durability and arrhythmia recurrence remain challenging [10–12]. The safety improvements accompanying irrigated catheter designs resulted in a shift away from tissue temperature as the primary metric of effective lesion delivery. This catheter paradigm triggered iterative surrogates to facilitate feedback on energy transfer (i.e., contact force, stability), lesion durability (i.e., ablation index, lesion-size index), and procedural efficiency (i.e., high-power short-duration (HPSD), inter-lesion distance) [13–19].

From the continued need for procedural improvement, a novel catheter (ground-up design) focused on accurate temperature feedback was developed [20, 21]. Through externally located thermocouples, incorporation of industrial diamond, and a low irrigation rate, the DiamondTemp ablation (DTA) system (Medtronic Inc, Minnesota, MN) has established a tip-tissue interface temperature accuracy of 2–4 °C [22]. Recent clinical successes have been demonstrated for atrial arrhythmias with ablation parameters akin to HPSD [23–27]. However, the data for ventricular or thicker tissue applications using a similar approach are less understood [28, 29]. This study sought to evaluate lesion morphologies across a broad range of ablation parameters (i.e., temperature set-points and application duration) to better understand system performance for a range of tissue thicknesses.

## Methods

### Ablation system

The study was conducted using the DTA system FASTR RF generator (model CEDTG200), which has been previously described [21, 25]. In brief, the temperature-controlled system takes readings every 20ms from 6 external thermocouples; 3 located on the distal tip and 3 proximal to the RF electrode. Upon reaching the programmed temperature set-point (default 60 °C), the generator automatically modulates the power to maintain the tip-tissue temperature. Additionally, the catheter 4.1 mm split-tip design contains a chemical vapor deposition diamond network which allows for more efficient thermal transfer properties; ultimately allowing for a constant 8ml/min irrigation rate and accurate temperature measurements at the tip-tissue interface. The FASTR RF generator is the second-generation temperature-controlled system and incorporates an increased power modulation responsiveness, ramping to 50 W (default upper limit) within 1 s. In this experimental setup, the temperature set-point and the application duration were varied to create a range of lesions, and all other system properties were held constant, based on the device labeling.

### *Ex vivo* model

The *ex vivo* setup consisted of a ~20 L tank filled with 10 L of normal saline, and salt or water was added to adjust to a floating catheter impedance of 100–120 Ω. A continuous-flow pump and heating circuit were placed in the tank to heat the solution to 37 °C. An indifferent electrode was placed on the opposite end of the tank from the tissue sample, at a distance between 8 and 12 inches.

The DTA system was set up in accordance with the device labeling. The power limit was set to 50 W with an irrigation rate of 8 ml/min during RF delivery. Power modulation is predicated upon the tip-tissue temperature reaching the programmed temperature set-point. Applications were grouped by setting the temperature set-point to 50 °C and 60 °C (Group_50 and Group_60, respectively). With a lower temperature set-point, power modulation is expected to trigger earlier, potentially impacting lesion morphologies. The application duration varied between pre-determined values ranging from 5 to 120 s. All applications were delivered in a perpendicular catheter orientation.

Freshly excised porcine cardiac tissue was obtained, and the right ventricles were sectioned for use. Prior to ablation, each section was prepped, placed in the tank, and allowed to acclimate for approximately 5 min. Ablations were delivered in a randomized order, leaving adequate space between applications for measurements. If a steam pop occurred, the catheter was repositioned to unablated tissue and the application was repeated, and two sequential steam pops resulted in a failed attempt.

### Visualization and comparison of lesion data

The methods for visualization and measurement have been previously described [30–32]. In brief, upon completion of a given tissue sample, the tissue was placed in a warmed 2,3,5-triphenyltetrazolium chloride stain for lesion evaluation. The surface width and orthogonal length were measured for each lesion. All lesions were then dissected through their width and re-stained for cross-sectional measurements including overall lesion depth, maximum width, and depth at maximum width.

### Statistics

Temperature data from the DTA system was evaluated for each individual application. The lesion dimensions were also categorized by their application settings. All continuous variables are represented as a mean ± standard deviation. Comparisons between groups were done using either an unpaired *t*-test or a one-way analysis of variance (ANOVA) for normally distributed variables. Post hoc pairwise comparison via Tukey’s test was performed in response to ANOVA significance, where a *p*-value of <0.05 was considered significant. Continuous variables were fit to regression models to describe lesion growth.

## Results

DTA applications were delivered in a randomized order across a series of bench experiments. In total, 280 applications were attempted, 141 in Group_50 and 139 in Group_60. Five (1.8%) applications resulted in steam pop and were removed from this analysis due to application disruption and tissue sample distortion. Two 90-s and three 120-s applications in Group_60 resulted in steam pops at an average of 61.1 s. The applications in Group_50 presented average temperatures, powers, and impedance drops of 49.2 ± 1.0 °C, 30.3 ± 7.6 W, and 15 + 8 Ω, respectively. In the Group_60 cohort, the same measures were 57.9 ± 2.6 °C, 39.9 ± 7.5 W, and 18 ± 7 Ω, respectively.

### 50 °C temperature set-point applications

**Fig. 1:**
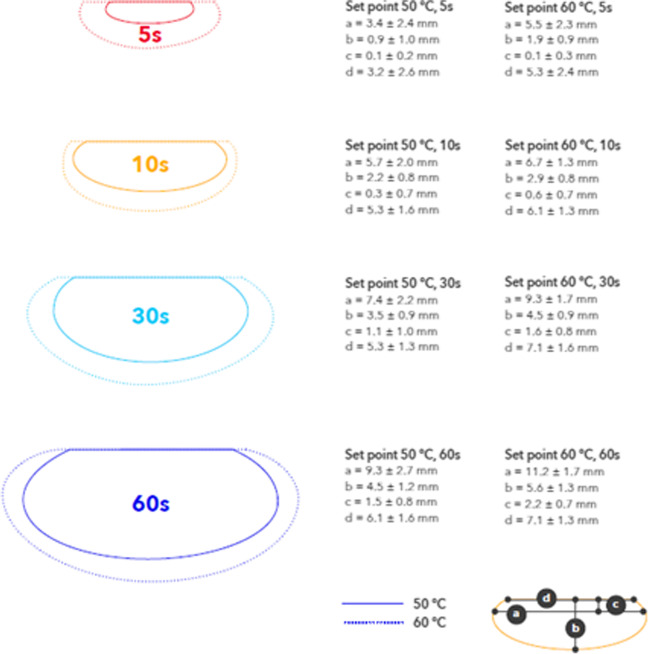
Lesion morphology comparison. Average lesion dimensions from the DiamondTemp applications using the 50 °C and 60 °C temperature set-points with durations ranging from 5 to 60s

In Group_50 (Table 1, Fig. 1), lesion depths increased from 0.9 ± 1.0 mm at 5 s to 4.5 ± 1.2 mm at 60 s, with no additional significant increase for 90- or 120-s applications. Similarly, maximum width increased with ablation duration until 60 s, with no significant depth increases beyond a 60-s application. Surface width plateaued after 10 s (5.3 ± 1.6 mm), at which time, the depth (at maximum width) continued to increase from 0.3 ± 0.7 mm to 2.3 ± 1.0 mm at 120 s. As the application durations extended, the temperature set-point was reached allowing for more power titration. In 5-s applications, average power was 34.9 ± 7.9 W, whereas in 30- and 60-s applications, the power averaged 29.7 ± 4.4 W and 29.4 ± 7.4 W respectively.

**Table 1:** Lesion morphologies and generator parameters from DiamondTemp applications delivered with the temperature set-point of 50 °C

Group_50							
Application duration (s)	Depth (mm)	Surface width (mm)	Max width (mm)	Depth at max width (mm)	Avg temp (°C)	Avg power (W)	Impedance drop (ohms)
5 (*n*=17)	0.9 ± 1.0	3.2 ± 2.6	3.4 ± 2.4	0.1 ± 0.2	47.4 ± 0.6	34.9 ± 7.9	10 ± 8
10 (*n*=17)	2.2 ± 0.8	5.3 ± 1.6	5.7 ± 2.0	0.3 ± 0.7	48.8 ± 0.2	34.2 ± 4.9	12 ± 8
15 (*n*=19)	2.5 ± 0.7	4.9 ± 1.7	6.1 ± 2.0	0.5 ± 0.6	48.9 ± 1.2	33.1 ± 8.1	15 ± 8
30 (*n*=18)	3.5 ± 0.9	5.3 ± 1.3	7.4 ± 2.2	1.1 ± 1.0	49.7 ± 0.0	29.7 ± 4.4	15 ± 9
45 (*n*=19)	3.3 ± 1.1	5.0 ± 1.6	7.3 ± 2.1	1.0 ± 0.8	49.6 ± 0.8	29.6 ± 8.0	16 ± 8
60 (*n*=16)	4.5 ± 1.2	6.1 ± 1.6	9.3 ± 2.7	1.5 ± 0.8	49.7 ± 0.4	29.4 ± 7.4	17 ± 8
90 (*n*=18)	4.4 ± 0.9	5.8 ± 1.5	8.4 ± 2.7	1.8 ± 0.9	49.9 ± 0.0	24.4 ± 4.8	16 ± 6
120 (*n*=17)	4.4 ± 0.8	5.6 ± 1.5	10.0 ± 2.6	2.3 ± 1.0	49.8 ± 0.3	27.3 ± 9.3	17 ± 8

### 60 °C temperature set-point applications

In Group_60 (Table 2, Fig. 1), lesion depths ranged from 1.9 ± 0.9 mm at 5 s to 5.6 ± 1.3 mm at 60 s, with no significant increase beyond a 60-s application. Additionally, it should be noted there was no significant change in depth between the 30-, 45-, and 60-s applications. However, significant increases were observed when comparing 30- and 45-s applications to 90- and 120-s applications. Surface width plateaued after 10 s (6.1 ± 1.3 mm), and at this time, the depth (at maximum width) continued to increase from 0.6 ± 0.7 mm to 2.9 ± 1.2 mm at 120 s. As the application durations extended, the temperature set-point was reached allowing more power titration, though to a lesser degree than in Group_50. In 5-s applications, the average power was 46.9 ± 3.9 W, whereas in 30- and 60-s applications, the power averaged 38.8 ± 6.8 W and 37.1 ± 5.1 W respectively.

**Table 2:** Lesion morphologies and generator parameters from DiamondTemp applications delivered with the temperature set-point of 60 °C

Group_60							
Application duration (s)	Depth (mm)	Surface width (mm)	Max width (mm)	Depth at max width (mm)	Avg temp (°C)	Avg power (W)	Impedance drop (ohms)
5 (*n*=16)	1.9 ± 0.9	5.3 ± 2.4	5.5 ± 2.3	0.1 ± 0.3	54.2 ± 3.1	46.9 ± 3.9	−13 ± 6
10 (*n*=18)	2.9 ± 0.8	6.1 ± 1.3	6.7 ± 1.3	0.6 ± 0.7	55.9 ± 2.6	46.1 ± 3.8	−16 ± 9
15 (*n*=16)	3.1 ± 0.8	6.1 ± 1.5	7.8 ± 2.2	1.0 ± 0.7	56.9 ± 2.4	44.9 ± 6.4	−15 ± 6
30 (*n*=18)	4.5 ± 0.9	7.1 ± 1.6	9.3 ± 1.7	1.6 ± 0.8	59.0 ± 0.6	38.8 ± 6.8	−20 ± 7
45 (*n*=17)	4.6 ± 0.9	6.3 ± 1.1	9.3 ± 2.6	1.8 ± 1.0	59.0 ± 1.1	36.6 ± 8.0	−21 ± 6
60 (*n*=17)	5.6 ± 1.3	7.1 ± 1.3	11.2 ± 1.7	2.2 ± 0.7	59.3 ± 0.6	37.1 ± 5.1	−20 ± 7
90 (*n*=16)	6.1 ± 1.5	6.5 ± 1.1	11.3 ± 2.7	2.5 ± 1.0	59.5 ± 0.5	35.2 ± 5.4	−20 ± 6
120 (*n*=16)	5.9 ± 1.4	7.6 ± 1.4	11.8 ± 2.3	2.9 ± 1.2	59.5 ± 0.7	33.4 ± 5.9	−22 ± 7

### Comparison of lesion morphologies for temperature set-points of 50 and 60 °C

**Fig. 2:**
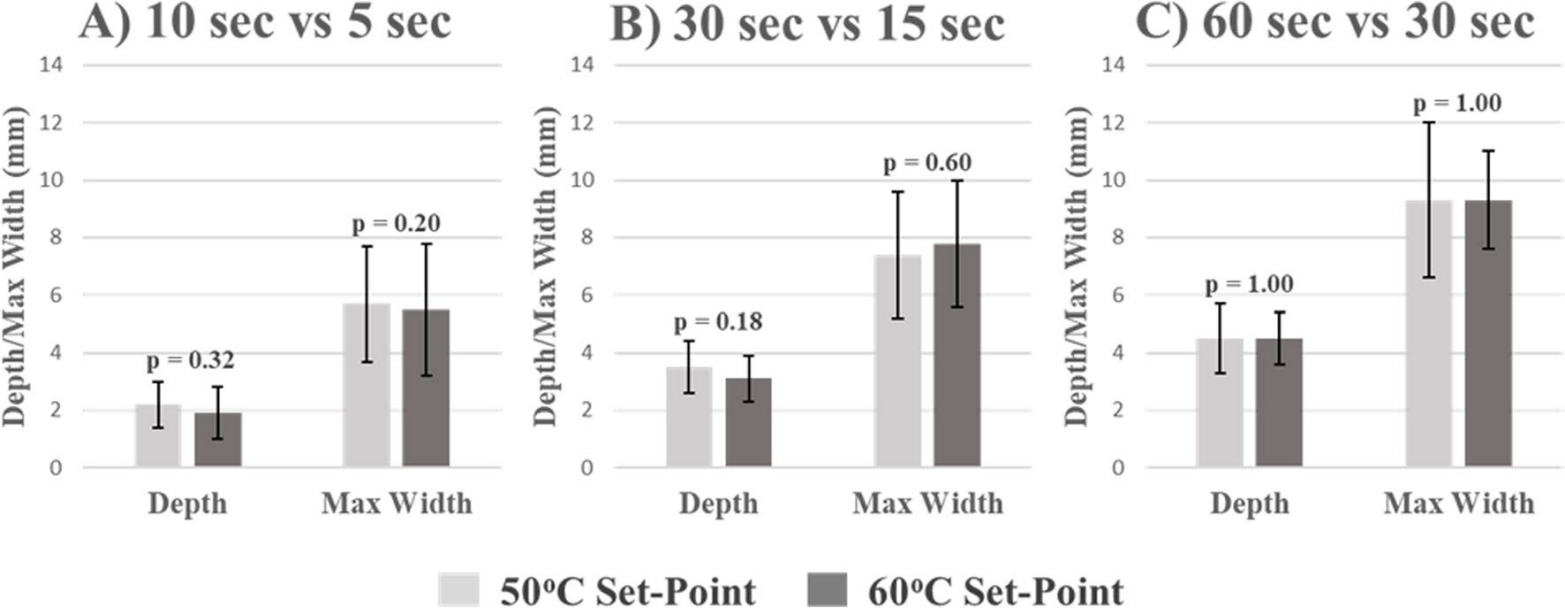
Lesion morphology according to temperature set-point and duration. The lesion dimensions for the different temperature set-points show consistency when the duration is doubled. Panel **A** shows the dimensions from the 5-s applications with the 60 °C temperature set-point and the 10-s applications with the 50 °C temperature set-point. Panel **B** shows the dimensions from the 15-s applications with the 60 °C temperature set-point and the 30-s applications with the 50 °C temperature set-point. Panel **C** shows the dimensions from the 30-s applications with the 60 °C temperature set-point and the 60-s applications with the 50 °C temperature set-point

Applications longer than 60 s in both Group_50 and Group_60 showed no marked increase in lesion morphology. Comparative analyses excluded the 90- and 120-s applications to focus on clinically relevant application durations. Applications in Group_60 generally produced deeper and wider lesions than those in Group_50. However, when comparing the lesion morphologies between Group_50 and Group_60, a pattern emerges. When application durations for Group_50 were twice the duration of Group_60, there were no significant differences in lesion depth and maximum width (Fig. 2a–c). This relationship remains consistent across the range of application durations: 10 s vs. 5 s, 30 s vs. 15 s, and 60 s vs. 30 s for Group_50 and Group_60. When adjusting the temperature set-point to 50 °C, the ability to deliver a comparable lesion took twice as long. In making this adjustment to the system, the average power and average temperature are also markedly different between Group_50 and Group_60.

### Time after temperature 60 °C analysis

**Fig. 3:**
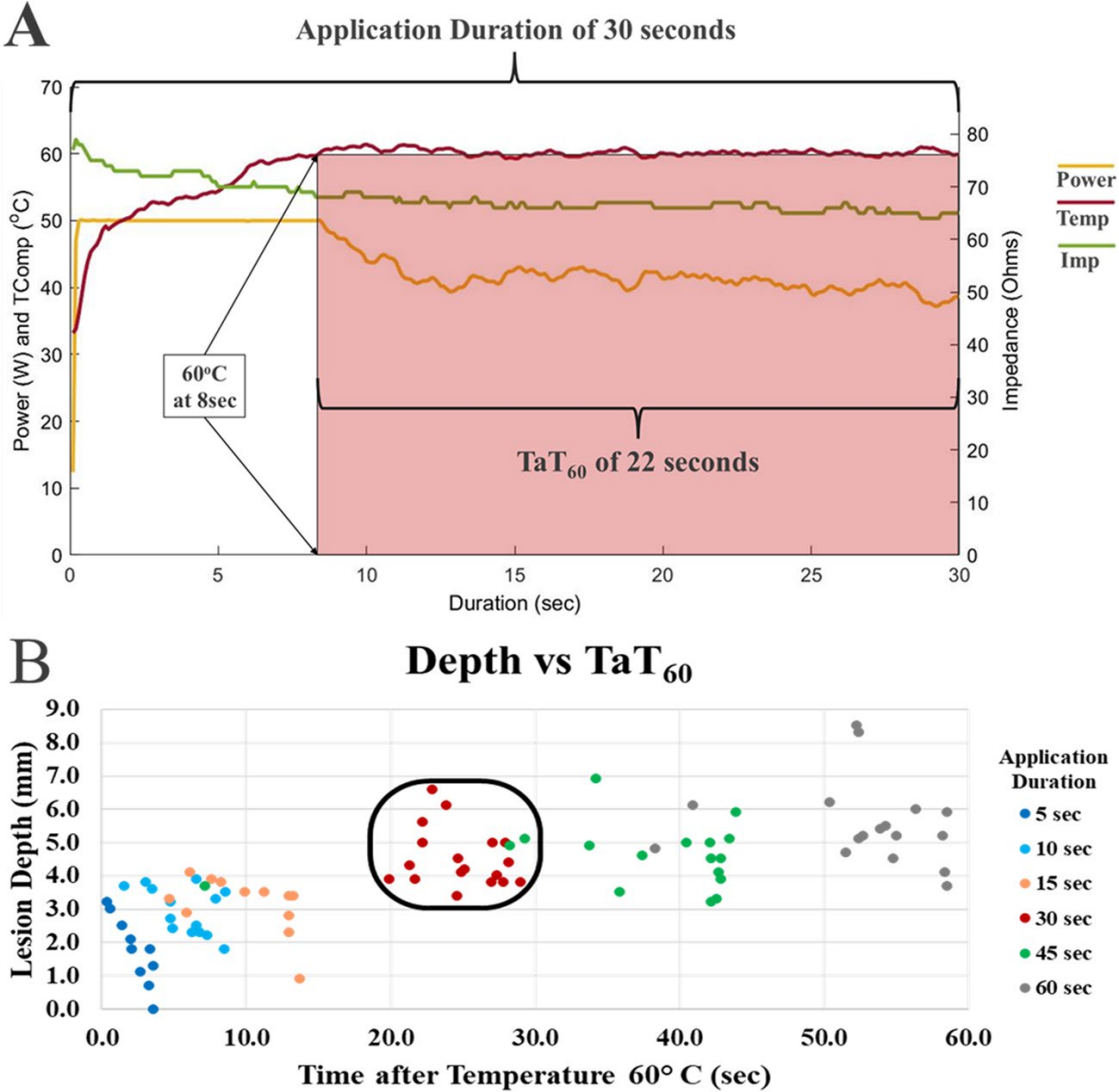
Time after temperature 60 °C and patterns of lesion depth. Panel **A** shows a DTA application which reaches the temperature set-point (60 °C) at 8s and modulates the power accordingly for the remainder of the 30-s application. The application has a TaT_60_ of 22 s. Panel **B** shows by analyzing the time each application reached 60 °C, a clustering of lesion depths occurs and plateaus. The rectangle represents potential target durations for ventricular applications based on maximizing the depth while minimizing the risk of overheating the tissue

Additional analysis of the Group_60 data indicated a strong relationship with time after reaching a tip-tissue temperature of 60 °C (time after temperature 60 °C—denoted TaT_60_) and total lesion depth, independent of the total application duration (Fig. 3A). The concept of “time after temperature 60 °C” is illustrated in Fig. 3A. During this single 30-s application, a tip-tissue temperature of 60 °C was reached after 8 s from RF onset; thus, the measured TaT_60_ in this application was 22 s. When TaT_60_ measured under 5.0 s, the average depth was 2.4 ± 1.1 mm. Lesion depth exhibited a non-linear relationship and the rate of lesion growth slowed beyond 20–30 s TaT_60_ where depth averaged 4.6 ± 0.8 mm (*n*=19) (Fig. 3B). Depth measured 5.1 ± 1.2 (*n*=31) when TaT_60_ was between 30 and 60 s, which was no different to the 20–30 s TaT_60_ lesions (*p*=0.11).

## Discussion

Temperature-controlled, power-modulated irrigated RF ablation with the DiamondTemp ablation system has been reported for both atrial and ventricular ablation [24, 26, 27, 29]. The focus of this study utilizing an *ex vivo* porcine cardiac model was to explore lesion morphologies at temperature set-points of 50 °C or 60 °C and at a range of ablation durations that may aid in developing dosing strategies. The key findings from this study were as follows:

1. In both study groups, lesion depth plateaus after 60 s and location of maximum lesion width continues to deepen as application durations extend.
2. Lesion morphologies in Group_60 were generally larger than those of the same duration in Group_50. Group_60 applications created similar-sized lesions in half the time compared to Group_50.
3. “Time after temperature 60 °C”—TaT_60_ is a promising parameter to determine lesion depth. Lesion depth plateaus after reaching a temperature of 60 °C for 20–30 s.

### Lesion biophysics with temperature-controlled RF

With a temperature-controlled system, operators can maximize resistive heating to create wide lesions similar to a HPSD approach, while allowing power modulation to maintain tissue temperature within a therapeutic range. The findings of this study show that lesion morphologies (depth and maximum width) in the Group_50 and Group_60 groups incrementally expanded between 5- and 60-s applications and showed no substantial increase with 90- or 120-s applications. Group_50 applications demonstrated incremental lesion depths from 0.9 ± 1.0 mm at 5 s to 4.5 ± 1.2 mm at 60 s, with no significant increases beyond a 60-s application. Similarly, the maximum width also increased with application duration until 60 s. In the Group_60 applications, lesion depths ranged from 1.9 ± 0.9 mm at 5 s to 5.6 ± 1.3 mm at 60 s, with no significant increases beyond a 60-s application. Similarly, maximum width also increased with ablation duration until 60 s. Dr. Winkle’s assertion that resistive heating transitions to conductive heating within the first 10 s [33] is further supported by the shift in lesion shape as the maximum width surpasses the surface width and continued deepening of the depth of maximum width between 5 and 10-s applications. In a study by Verma and colleagues [21], they assessed lesion morphology in multiple test models with a temperature set-point of 60 °C. In their work, a porcine thigh model demonstrated average lesion depths of 3.2, 4.4, and 5.2 mm for 5-, 10-, and 15-s applications, respectively. While reaching a similar maximum temperature, these dimensions were larger than the findings in the present study, suggesting the model may play a role in overall performance. The DTA system was used in an *in vivo* canine model to deliver discrete lesions in all chambers of the heart. Using similar application parameters, ventricular lesions with durations of 17.9 ± 3.4 s achieved more comparable lesion depths of 6.0 ± 1.6 mm [28]. The observed differences in the lesion biophysics may be related to the TaT_60_ of the applications. Analysis of 15-s applications in Group_60 resulted in lesion depths of 3.1 ± 0.8 mm. However, applications with TaT_60_ of 20 s resulted in lesion depths of 4.6 ± 0.8 mm.

Recently, reports of a novel ablation system utilizing an ultra-HPSD (90W × 4s) approach have surfaced, demonstrating predominantly transmural atrial lesions with widths ranging from 5 to 10 mm [34, 35]. The ventricular lesions presented with this ultra-HPSD approach were reported to be shallow (2.7–3.8 mm) and wide (6.9–7.8 mm) in nature. More recently, a modified ultra-HPSD 10-s step-down approach (90W × 4s titrated to 50W × 6s) demonstrated lesion depths of 3.9 mm and widths of 7.7 mm [36]. The lesion characteristics from the present study stand up considerably against the ultra-HPSD approach with the addition of a maximum power delivery of 50 W. Furthermore, as longer application times are required to generate deeper lesions, the DTA system delivers high power with power titration reducing the risk of tissue superheating. This system accurately measures the temperature at the tip-tissue interface and modulates power accordingly. This may slow the rate of temperature change within the tissue but does not inhibit the rise in inter-tissue temperature. In this study of 280 temperature-controlled RF applications, only five steam pops (1.8%) were observed with an average duration to pop of greater than 60 s.

Contact force has proven to be influential for lesion formation in power-controlled radiofrequency ablation [13, 14]. The role contact force has on lesion formation with DTA may differ based on application duration. Lesion dimensions from 5 to 15-s applications with the DTA system have been shown to be independent of the level of applied force [21]. Counter to this, Sasaki et al. [37] demonstrated increased applied force positively correlated with lesion dimensions with 6-s applications; however, these measurements appear to be inclusive of steam pop events. Interestingly, this study also presented a non-linear relationship between lesion dimensions as application duration lengthens. Our results support these findings that the ability to titrate power based on an accurate tip-tissue temperature will ultimately result in consistent lesion formation across a range of clinically relevant applied forces. Temperature-controlled ablation produces similar-sized lesions to power-controlled ablations and increasing contact force in the latter is associated only with an increased risk of steam pop [21, 34, 37].

### Clinical implications

The DTA technology is predicated on modulating power once the set-point temperature is achieved. This is enabled through the enhanced thermal diffusivity of the industrial diamond elements embedded in the distal portion of the catheter, which allows for highly accurate tip-tissue temperature readings [20, 22]. From these accurate real-time temperature readings, the power is then modulated, thus allowing the resistive heating to remain in the optimal therapeutic range. Our findings show that at temperature set-points of both 50 and 60 °C, increasing ablation duration will provide greater lesion width and depth with a plateauing effect for depth at 60 s. In previous DTA atrial fibrillation clinical trials, ablation recommendations were a temperature set-point of 60 °C, and ablation termination guidance was 3 to 5 s beyond electrogram amplitude attenuation of 75 to 80% [20, 23–25]. During the DIAMOND-AF trial, the average application duration, with the first-generation DTA system, was 14.7 ± 5.3 s resulting in a 79.1% freedom from recurrence of any atrial arrhythmia during the 12-month follow-up. Similarly, in the FASTR-AF study, which introduced the updated generator with increased power modulation responsiveness, the average application duration was 10.3 ± 3.3 s, resulting in freedom from arrhythmia recurrence of 73.4%. By using the same ablation parameters, the present study shows that 10-s applications created lesion depths of 2.9 ± 0.8 mm and maximum lesion widths of 6.7 ± 1.3 mm, which fall well within the requirements for atrial transmurality. While shorter applications resulted in lesion depths better suited for atrial tissue, further testing should be conducted to evaluate the effectiveness of TaT_60_ as a metric for atrial applications.

Al Sheikhli and colleagues [29] reported the feasibility of using the DTA catheter system for ventricular tachycardia ablation in ischemic cardiomyopathy patients. Ablation was delivered with a temperature set-point of 60 °C for a maximum of 60 s and limited to bipolar electrogram amplitude attenuation of 75 to 80% or impedance reduction of no more than 20 Ω. Applying these ablation parameters, the present study findings would suggest lesion depths of 5.6 ± 1.3 mm, albeit in healthy tissue. The authors reported a single steam pop with a 44-s ablation lesion where the maximum temperature reached was 60 °C with an impedance reduction of 20 Ω. Interestingly, the maximum temperature was reached within the initial 10 s, and thus 34 s of ablation was delivered beyond reaching the pre-set temperature. The present study demonstrated a plateau in depth when TaT_60_ reaches 20 s. It is unknown if the steam pop would have been avoided had the ablation duration been limited to TaT_60_ of 20 s and if the necessary lesion size had been achieved.

Establishing stopping criteria for energy delivery is paramount for lesion formation to not extend beyond the cardiac tissue and to minimize tissue overheating. The plateau in depth identifies a point of diminishing return for continued RF delivery in ventricular tissue. Utilizing TaT_60_ criteria may also serve as a function of titrating RF time and thus potentially increasing efficiency in ventricular ablation procedures. Further clinical studies are ongoing applying this tailored DiamondTemp ablation approach where individual ablation application is based on the TaT_60_ duration.

## Limitations

The *ex vivo* nature of this study may limit the ability to translate our findings to clinical outcomes. Experiments conducted on excised myocardial tissue lack cardiac motion and blood flow patterns that would be present in a clinical scenario. In this study, it was observed that when the temperature set-point of 50 °C or 60 °C was reached, as happened in most instances, it was maintained for the remainder of the application. In addition, the absence of perfused viable myocardium has an impact on intramuscular cooling as well as pre-ablation impedance values. This can have an impact on heat transfer into the tissue and may have an impact on lesion formation along with temperature recordings from the tip-tissue interface. Finally, slight variations in relative catheter contact and orientation at the tip-tissue interface can limit the rate at which temperature increases throughout the ablation, and the temperature set-point may not always be achieved. These limitations do not change the relationship between the measured temperatures presented in this study and the correlated lesion morphologies.

## Conclusion

The DTA system rapidly creates wide lesions with lesion depth increasing over time with application durations up to 60 s. The introduction of “time after temperature 60 °C” demonstrates a potential clinical target for a stopping criterion for ventricular applications. With a temperature-controlled system, operators can maximize the resistive heating to create wide lesions, while allowing power modulation to maintain tissue temperature within a therapeutic range.

## Abbreviations

DTA: DiamondTemp ablation
HPSD: High-power short-duration
RF: Radiofrequency
TaT_60_: Time after temperature 60 °C
